# Testing and Management of Iron Overload After Genetic Screening–Identified Hemochromatosis

**DOI:** 10.1001/jamanetworkopen.2023.38995

**Published:** 2023-10-23

**Authors:** Juliann M. Savatt, Alicia Johns, Marci L. B. Schwartz, Whitney S. McDonald, Zachary M. Salvati, Nicole M. Oritz, Max Masnick, Kathryn Hatchell, Jing Hao, Adam H. Buchanan, Marc S. Williams

**Affiliations:** 1 Department of Genomic Health, Geisinger, Danville, Pennsylvania; 2 Department of Population Health Sciences, Geisinger, Danville, Pennsylvania; 3 Ted Rogers Centre for Heart Research, Cardiac Genome Clinic, The Hospital for Sick Children, Toronto, Ontario, Canada; 4 Division of Clinical and Metabolic Genetics, The Hospital for Sick Children, Toronto, Ontario, Canada

## Abstract

**Importance:**

*HFE* gene–associated hereditary hemochromatosis type 1 (HH1) is underdiagnosed, resulting in missed opportunities for preventing morbidity and mortality.

**Objective:**

To assess whether screening for p.Cys282Tyr homozygosity is associated with recognition and management of asymptomatic iron overload.

**Design, Setting, and Participants:**

This cross-sectional study obtained data from the Geisinger MyCode Community Health Initiative, a biobank of biological samples and linked electronic health record data from a rural, integrated health care system. Participants included those who received a p.Cys282Tyr homozygous result via genomic screening (MyCode identified), had previously diagnosed HH1 (clinically identified), and those negative for p.Cys282Tyr homozygosity between 2017 and 2018. Data were analyzed from April 2020 to August 2023.

**Exposure:**

Disclosure of a p.Cys282Tyr homozygous result.

**Main Outcomes and Measures:**

Postdisclosure management and *HFE-*associated phenotypes in MyCode-identified participants were analyzed. Rates of *HFE-*associated phenotypes in MyCode-identified participants were compared with those of clinically identified participants. Relevant laboratory values and rates of laboratory iron overload among participants negative for p.Cys282Tyr homozygosity were compared with those of MyCode-identified participants.

**Results:**

A total of 86 601 participants had available exome sequences at the time of analysis, of whom 52 994 (61.4%) were assigned female at birth, and the median (IQR) age was 62.0 (47.0-73.0) years. *HFE* p.Cys282Tyr homozygosity was disclosed to 201 participants, of whom 57 (28.4%) had a prior clinical HH1 diagnosis, leaving 144 participants who learned of their status through screening. There were 86 300 individuals negative for p.Cys282Tyr homozygosity. After result disclosure, among MyCode-identified participants, 99 (68.8%) had a recommended laboratory test and 36 (69.2%) with laboratory or liver biopsy evidence of iron overload began phlebotomy or chelation. Fifty-three (36.8%) had iron overload; rates of laboratory iron overload were higher in MyCode-identified participants than participants negative for p.Cys282Tyr homozygosity (females: 34.1% vs 2.1%, *P* < .001; males: 39.0% vs 2.9%, *P* < .001). Iron overload (females: 34.1% vs 79.3%, *P* < .001; males: 40.7% vs 67.9%, *P* = .02) and some liver-associated phenotypes were observed at lower frequencies in MyCode-identified participants compared with clinically identified individuals.

**Conclusions and Relevance:**

Results of this cross-sectional study showed the ability of genomic screening to identify undiagnosed iron overload and encourage relevant management, suggesting the potential benefit of population screening for *HFE* p.Cys282Tyr homozygosity. Further studies are needed to examine the implications of genomic screening for health outcomes and cost-effectiveness.

## Key Points

**Question:**

Is identification of *HFE* p.Cys282Tyr homozygosity among individuals in an unselected health care system population associated with recognition of asymptomatic iron overload and prompt management?

**Findings:**

In this cross-sectional study of 144 participants with genomic screening–identified p.Cys282Tyr homozygosity, 36.8% had clinically unrecognized evidence of iron overload. Of those previously unaware of their *HFE* p.Cys282Tyr homozygosity results with evidence of iron overload, 69.2% completed interventions known to reduce the risk of end organ damage.

**Meaning:**

Findings of this study suggest that the return of homozygous *HFE* p.Cys282Tyr variant results should be evaluated for potential inclusion in population genomic screening.

## Teaser

This cross-sectional study assesses the rates of recognition and management of asymptomatic iron overload after identification of hereditary hemochromatosis type 1 via genetic screening.

## Introduction

Hereditary hemochromatosis type 1 (HH1; OMIM 235200^1^) predisposes to iron overload with iron accumulation in the liver, heart, and pancreas. Without treatment, iron overload can result in morbidity and mortality associated with end-stage liver disease (ESLD, including fibrosis, cirrhosis, and hepatocellular carcinoma), cardiomyopathy, and diabetes. Iron overload can be effectively treated and, if initiated early, can reverse or prevent organ damage.

Hereditary hemochromatosis type 1 is caused by variants in the *HFE* gene (OMIM 613609).^2,3^ The c.845G>A missense variant (p.Cys282Tyr) is associated with the highest risk for iron overload when present on both alleles (p.Cys282Tyr homozygote).^4^ Hereditary hemochromatosis type 1 has variable prevalence in ancestral groups but is the most common genetic disease in Northern Europeans, with an estimated prevalence of 1 in 150 to 400 people.^4,5,6,7^ Population screening for HH1 has been considered by several professional entities, including the US Preventive Services Task Force, American College of Physicians, and Centers for Disease Control and Prevention (CDC), and representatives of gastroenterology specialty societies. Each entity has either found insufficient evidence to support such screening or has recommended against it.^8,9,10,11,12^

A review of clinical penetrance in p.Cys282Tyr homozygotes estimated that 10% of males were likely to develop severe liver disease if iron overload was not identified and treated prior to irreversible liver damage.^13^ A study from the UK Biobank,^14^ with approximately 3000 p.Cys282Tyr homozygotes, detected substantial increases in the prevalence of hemochromatosis, liver disease, rheumatoid arthritis, osteoarthritis, and diabetes in individuals with the variant vs controls without the variant. Further analyses found an increased risk for hepatic malignant neoplasm in male homozygotes^15^ and, in a subset of participants aged 65 to 70 years, identified increased prevalence of frailty, sarcopenia, and chronic pain.^16^

Although these data suggest that p.Cys282Tyr homozygosity is associated with increased risk for end organ damage in broader cohorts, no published population screening studies have returned *HFE* results to participants. Consequently, comparisons of disease severity in individuals with clinically identified vs genomic screening–identified p.Cys282Tyr homozygosity are limited, and downstream impact of screening is undetermined.

Recently, p.Cys282Tyr homozygosity was added to the American College of Medical Genetics and Genomics (ACMG) Secondary Findings list for assessment and return on indication-based sequencing.^17^ Currently, the Centers for Disease Control and Prevention Office of Genomics and Precision Public Health is considering adding p.Cys282Tyr homozygosity to its tier 1 list, defined as genomic interventions that have substantial potential to benefit public health.^11^ If broader screening for p.Cys282Tyr homozygosity is to be considered, evidence is needed demonstrating the ability of genomic screening to identify unrecognized iron overload before clinical diagnosis and documentation of treatment efficacy.

To identify the utility of population screening for p.Cys282Tyr homozygosity, we measured iron overload–associated diagnoses and postdisclosure health behaviors in individuals who were identified via genomic screening, and we compared the rates of *HFE-*associated phenotypes among individuals with genomic screening–identified positive p.Cys282Tyr homozygosity results (indicating presence of the variant in the homozygous state), with clinically diagnosed HH1, and among those negative for p.Cys282Tyr homozygosity (indicating absence of homozygosity for the variant).

## Methods

### Study Design, Setting, and Participants

This retrospective cross-sectional study focused on participants who received p.Cys282Tyr homozygous results through the Geisinger MyCode Community Health Initiative (MyCode) or had their exome assessed and were found to have negative p.Cys282Tyr homozygosity prior to the COVID-19 pandemic (between 2017 and 2018). The Geisinger Institutional Review Board approved MyCode and this study. All participants provided written informed consent. We followed the Strengthening the Reporting of Observational Studies in Epidemiology (STROBE) reporting guideline.

MyCode is a biobank of biological samples and linked electronic health record (EHR) data from over 333 000 participants recruited regardless of their phenotype.^18,19,20^ Participants consent to broad health-related research, including genetic analysis, and a subset of participants’ exomes have been sequenced as part of a collaboration with Regeneron Genetics Center.^21^ Since 2013, MyCode participants have consented to receive disclosure of medically actionable results.^22,23,24^ Aggregated variant files are filtered and reviewed for pathogenic or likely pathogenic variants in actionable genes that are designated in the ACMG Secondary Findings V3.1, including *HFE* p.Cys282Tyr homozygosity.^25,26^ Participants are eligible to receive results if their variants are confirmed as pathogenic or likely pathogenic by a Clinical Laboratory Improvement Amendments–certified clinical laboratory.^23^ Participants receiving results are offered a complimentary follow-up with a genetics professional to review the result and discuss associated management.^22,23^

Participants with p.Cys282Tyr homozygosity who received their positive results via initial MyCode screening were grouped as MyCode identified. Participants with previously clinically diagnosed HH1 were grouped as clinically identified.

### Clinical Data Collection and Outcome Measures

Clinicians familiar with HH1 adapted the eMERGE Network abstraction form^27^ to delineate variables of interest (eMethods in Supplement 1). For p.Cys282Tyr homozygotes, automated queries of relevant diagnosis codes (eMethods in Supplement 1), procedures, and laboratory values from the Geisinger EHR were completed and deposited in the Geisinger Research Electronic Data Capture tool.^28,29^ Certified genetic counselors (J.M.S., M.L.B.S., Z.M.S., N.M.O.) completed dual manual EHR reviews between October 2019 and April 2020 to verify automated queries and collect unstructured data. Discrepancies were resolved through consensus and physician review (M.S.W.). Automated EHR queries of relevant laboratory values were completed for participants negative for p.Cys282Tyr homozygosity.

### Postdisclosure Health Behaviors and Laboratory Findings

Postdisclosure health behaviors examined in MyCode-identified participants included laboratory testing relevant to iron metabolism, liver biopsy, cardiac magnetic resonance imaging (MRI), phlebotomy, and chelation. For each behavior that was completed after disclosure, attribution to the *HFE* result disclosure was assessed using clinician documentation (eg, medical record notes) and diagnostic codes associated with orders in the EHR.

Laboratory test results relevant to iron metabolism that were available in the EHR were evaluated for each MyCode-identified participant. Each participant’s highest serum iron, serum ferritin, and transferrin saturation levels and lowest total iron-binding capacity (TIBC) level were collected and assessed for evidence of iron overload. Participants were considered to have iron overload if they met laboratory, imaging, or other criteria.

The laboratory criteria were transferrin saturation of 45% or greater and serum ferritin level of 300 ng/mL or greater in individuals assigned male at birth or 200 ng/mL or greater in individuals assigned female at birth^10,12^ (to convert serum ferritin to microgram per liter, multiply by 1.0); laboratory studies could be completed asynchronously. Imaging criteria were a radiology report explicitly referencing iron overload (eg, moderate hepatic iron deposition noted on MRI). Other criteria were iron overload on liver biopsy or clinical documentation of iron overload such as on the problem list, encounter diagnoses, clinician documentation, and scanned notes.

### Comparisons and Definitions

Iron metabolism laboratory values and laboratory iron overload rates were compared between MyCode-identified participants and participants negative for p.Cys282Tyr homozygosity. The *HFE-*associated phenotypes that were compared between MyCode-identified participants and clinically identified participants included iron overload, liver disease, and heart disease. Liver disease was defined as fibrosis, cirrhosis, nonalcoholic steatohepatitis and/or nonalcoholic fatty liver disease, abnormal results of a liver function test (≥2 elevated transaminase measurements in the EHR ≥3 months apart without an intervening normal value), or other documentation of chronic liver disease (eg, diagnosis of liver disease on the problem list). As a secondary research question exploring the synergistic effect of genomic and other liver disease risk factors, frequency of liver disease was assessed in MyCode-identified participants and clinically identified participants with or without liver disease risk factors, defined as chronic hepatitis C virus (HCV) infection and alcohol use disorder (AUD; mention of AUD in the EHR). Heart disease was defined as evidence of cardiomyopathy and/or heart failure documented in the EHR.

### Statistical Analysis

The null hypotheses were as follows: (1) there was no difference in iron overload and associated diseases between MyCode-identified participants and clinically identified participants or between MyCode-identified participants and participants negative for p.Cys282Tyr homozygosity, and (2) genetic result disclosure had no implications for health behaviors or process outcomes. Data were stratified into MyCode-identified participants, clinically identified participants, and participants negative for p.Cys282Tyr homozygosity. Participants were further stratified based on sex assigned at birth. Any variable that was not in the EHR was treated as not performed. Categorical variables were described using frequency (percentage), and continuous variables were described using median (IQR). Pearson χ^2^ or Fisher exact tests and Wilcoxon rank sum tests were used to compare differences between groups in categorical and continuous variables, respectively.

Demographic characteristics of participants in the 3 groups were compared. Race and ethnicity were collected from the EHR and included the categories Black or African American, Hispanic or Latino, White, Other (including American Indian or Alaska Native, Asian, Native Hawaiian or Other Pacific Islander), not Hispanic or Latino, and unknown. Data on race and ethnicity were collected and analyzed in this study to report the demographic characteristics of the 3 cohorts and to inform the generalizability of results.

Odds ratios (ORs) and 95% CIs were used to quantify the relative strength of the association between *HFE*-associated phenotype and method of identification (MyCode identified vs clinically identified) or negative status. Completion of certain laboratory tests relevant to iron metabolism before vs on or after result disclosure was compared, and the McNemar test was used to assess statistical significance. Significant differences were determined at α = .05. All analyses were conducted using SAS, version 9.4 (SAS Institute Inc). Data were analyzed from April 2020 to August 2023.

## Results

**Figure 1.**
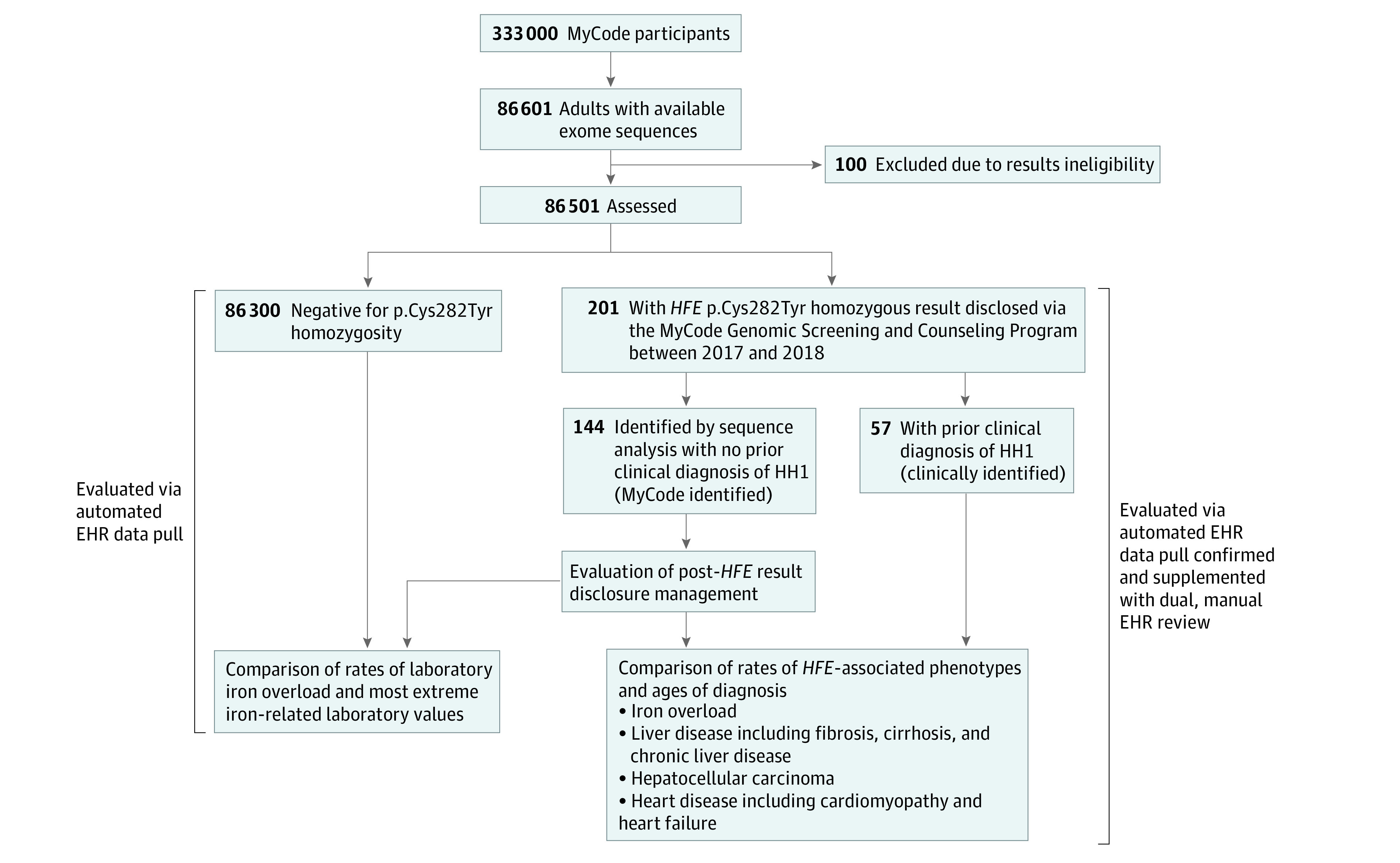
Flow Diagram of Study Design and Cohort Definitions EHR indicates electronic health record; HH1, hereditary hemochromatosis type 1.

After exclusion of 100 individuals who were ineligible to receive MyCode results, the analysis included 86 501 MyCode participants with available exome sequences (53 108 individuals assigned female at birth [61.4%], 33 388 individuals assigned male at birth [38.6%], and 5 individuals with unknown sex assigned at birth [.006%]) (Table 1). Participants had a median (IQR) age of 62.0 (47.0-73.0) years. *HFE* p.Cys282Tyr homozygosity was disclosed to 201 participants between 2017 and 2018, of whom 57 (28.4%) had a clinical diagnosis of HH1 prior to receiving their MyCode results (49 of whom had prior genetic testing) and were grouped as clinically identified participants. The remaining 144 participants (71.6%) learned of their status through MyCode screening and thus were grouped as MyCode-identified participants. There were 86 300 participants negative for p.Cys282Tyr homozygosity (Figure 1). Six MyCode-identified participants died between result disclosure and medical record review but were included in the analysis.

**Table 1.** Participant Demographics

Characteristic	MyCode participants with p.Cys282Tyr homozygous results, No. (%)		*P* value	MyCode participants negative for p.Cys282Tyr homozygosity, (n = 86 300), No. (%)	*P* value	
	MyCode identified (n = 144)	Clinically identified (n = 57)			Comparison to MyCode-identified participants	Comparison to clinically identified participants
Age at medical record review, median (IQR)	56.6 (41.6-71.8)	65.7 (52.3-70.3)	.07	62.0 (47.0-73.0)	.045^a^	.32
Sex assigned at birth						
Female	85 (59.0)	29 (50.9)	.29	52 994 (61.4)	.55^b^	.11^b^
Male	59 (41.0)	28 (49.1)		33 301 (38.6)		
Unknown	0	0		5 (<0.1)		
Ethnicity^c^						
Not Hispanic or Latino	144 (100)	57 (100)	NA	83 778 (97.1)	.02^a^	.42^a^
Unknown or Hispanic or Latino	0	0		2522 (2.9)		
Race^c^						
Black or African American	0	0	NA	1452 (1.7)	.25^b^	>.99^b^
White	144 (100)	57 (100)		84 189 (97.6)		
Other^d^	0	0		659 (0.8)		
Alive at medical record review	138 (95.8)	53 (93.0)	.47^a^	76 559 (88.7)	.007^a^	.31
Smoking status						
Current	24 (16.7)	9 (15.8)	>.99	14 086 (16.3)	.35^b^	.70^b^
Former	58 (40.3)	24 (42.1)		31 109 (36.0)		
Never	61 (42.4)	24 (42.1)		40 825 (47.3)		
Unknown	1 (0.7)	0		280 (0.3)		
Active alcohol drinker						
True	74 (51.4)	29 (50.9)	>.99^a^	40 090 (46.4)	.52^b^	.81^b^
False	69 (47.9)	28 (49.1)		45 071 (52.2)		
Not in medical record	1 (0.7)	0		1139 (1.3)		
AUD	18 (12.5)	6 (10.5)	.70	NA	NA	NA
Time from return of screening results, median (IQR), y	1.44 (1.35-1.51)	NA	NA	NA	NA	NA
Length of EHR, median (IQR), y	22.2 (14.6-25.5)	21.3 (15.4-23.6)	.72	17.0 (11.0-22.0)	<.001^a^	<.001^a^

Abbreviations: AUD, alcohol use disorder; EHR, electronic health record; NA, not applicable.

^a^ Calculated with Fisher exact test, with statistical significance at α = .05.

^b^ Calculated with Fisher exact test with Monte Carlo Estimation (seed = 1).

^c^ Race and ethnicity data were collected from the EHR.

^d^ Other included the following categories in the EHR: American Indian or Alaska Native (n = 109), Asian (n = 271), Native Hawaiian or Other Pacific Islander (n = 115), Other (n = 1), and Unknown (n = 163). The numbers for each category represent those of MyCode participants negative for p.Cys282Tyr homozygosity.

The median (IQR) time from result disclosure to review was 1.44 (1.35-1.51) years. Compared with participants negative for p.Cys282Tyr homozygosity, MyCode-identified participants were younger (median [IQR] age, 56.6 [41.6-71.8] years vs 62 [47.0-73.0] years; *P* = .045), included a higher proportion of non-Hispanic individuals (100% vs 97.1%; *P* = .02), had more longitudinal EHR data (median [IQR] length, 21.3 [15.4-23.6] years vs 17.0 [11.0-22.0] years; *P* = <.001), and had a larger proportion who were alive at medical record review (93.0% vs 88.7%; *P* = .007).

### Postdisclosure Findings 

After *HFE* result disclosure, 99 of 144 MyCode-identified participants (68.8%) had a serum ferritin (96 [66.7%]) or transferrin saturation (83 [57.6%]) test (Table 2), and 65 (45.1%) completed both tests. Most initial iron tests (84.4% [151 of 179]) could explicitly be attributed to the genetic result (Table 2). When comparing completion of these iron tests in MyCode-identified participants before vs after *HFE* result disclosure, all tests were completed at higher frequencies after result disclosure (Table 2). Patient-level completion of relevant laboratories is summarized in eTable 1 in Supplement 1.

**Table 2.** Postdisclosure *HFE*-Associated Management Among MyCode-Identified Participants (n = 144)

*HFE-*associated management	Total No. of MyCode-identified participants who ever completed (%)	Predisclosure, No. of participants (%)^a^	Postdisclosure, No. of participants (%)^a^	Days from postdisclosure, median (IQR)	Frequency attributed to *HFE* result disclosure, No. of participants (%)^b^	Completion of laboratory study predisclosure vs postdisclosure, *P* value
Serum iron (n = 144)	98 (68.1)	41 (41.8)	85 (86.7)	39.0 (15.0-165.0)	71 (83.5)^c^	<.001^d^^,^^e^
Serum ferritin (n = 144)^a^	104 (72.2)	39 (37.5)	96 (92.3)	27.0 (10.0-78.5)	83 (86.4)^c^	<.001^d^^,^^e^
Transferrin saturation (n = 144)^a^	95 (66.0)	32 (33.7)	83 (87.4)	42.0 (17.0-168.0)	68 (81.9)^c^	<.001^d^^,^^e^
TIBC (n = 144)	95 (66.0)	38 (40.0)	83 (87.4)	42.0 (17.0-168.0)	68 (81.9)^c^	<.001^d^^,^^e^
Liver panel (n = 144)	134 (93.1)	131 (97.8)	103 (76.9)	62.0 (27.0-178.0)	43 (41.7)^c^	<.001^d^^,^^f^^,^^e^
Phlebotomy (n = 144)	42 (29.2)	1 (2.4)	41 (97.6)	78.0 (43.0-180.0)	NA	NA
Phlebotomy, eligible patients (n = 52)^g^	36 (69.2)	1 (2.8)^h^	35 (97.2)	78.0 (42.0-180.0)	NA	NA
Chelation (n = 144)	1 (0.7)	0	1 (100)	19.0 (19.0-19.0)	NA	NA
Chelation, eligible patients (n = 52)^g^	1 (1.9)	0	1 (100)	19.0 (19.0-19.0)	NA	NA
Cardiac MRI (n = 144)	5 (3.5)	2 (40.0)	3 (60.0)	132.0 (57.0-211.0)	2 (66.7)	NA
Liver biopsy (n = 144)	9 (6.3)	4 (44.4)	5 (55.6)	200.0 (126.0-299.0)	5 (100)	NA

Abbreviations: MRI, magnetic resonance imaging; NA, not applicable; TIBC, total iron binding capacity.

^a^ Predisclosure and postdisclosure rates were reported as a proportion of the total number of participants who ever completed the risk management behavior.

^b^ Attribution of the postdisclosure initial management behavior was assessed and reported as a proportion of the total number of participants with that risk management behavior completed after result disclosure.

^c^ Additional participants had this study attributed to their result at a later time. The initial order was not attributed to the *HFE* p.Cys282Tyr homozygosity result and was therefore not counted as attributed to the result.

^d^ Calculated with McNemar test.

^e^ Statistically significant at α = .05.

^f^ Liver panels were completed less frequently after disclosure vs before disclosure.

^g^ Phlebotomy and chelation rates were reported as proportions of the 52 participants with iron overload identified in laboratory studies or on liver biopsy.

^h^ Before result disclosure, 1 MyCode-identified participant received phlebotomy secondary to a polycythemia diagnosis.

**Figure 2.**
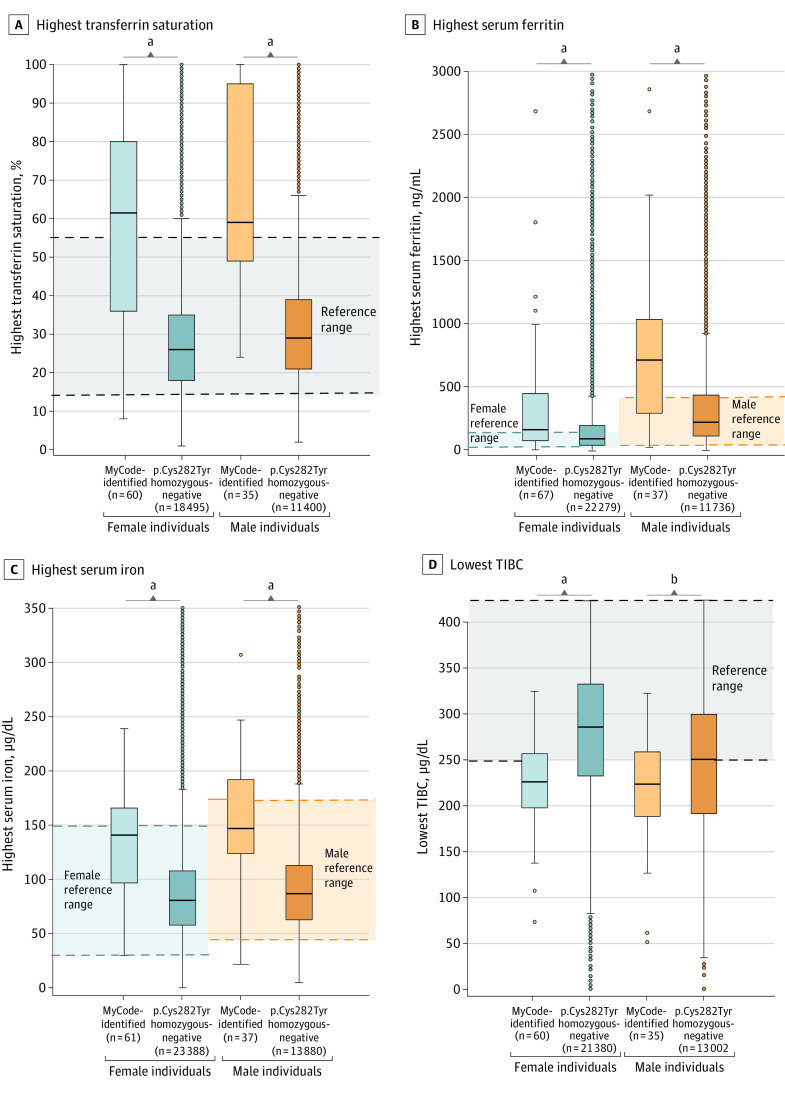
Most Extreme *HFE*-Associated Laboratory Values in MyCode-Identified Participants vs Participants Negative for p.Cys282Tyr Homozygosity Specific laboratory values were available in the electronic health record. Box plots show the median extreme value (horizontal line) and IQR for each laboratory study, stratified by sex assigned at birth. Outliers are noted as single data points. TIBC indicates total iron-binding capacity. SI conversion factor: To convert serum ferritin from nanogram per milliliter to microgram per liter, multiply by 1.0; serum iron and TIBC from microgram per deciliter to micromole per liter, multiply by 0.179. ^a^*P* < .001. ^b^*P* = .02.

The median highest values of serum ferritin and transferrin saturation and median lowest TIBC level were outside the reference ranges in both male and female MyCode-identified participants (Figure 2; eTable 2 in Supplement 2). Fifty-three of 144 MyCode-identified participants (36.8%) met criteria for laboratory or other iron overload, of which only 47 (88.7%) had such evidence after result disclosure (Table 3). Because iron overload penetrance is age related, age at time of result disclosure was analyzed. MyCode-identified female participants with evidence of iron overload were older than females without iron overload (median [IQR] age, 61.3 [51.1-69.9] years vs 46.2 [35.1-67.9] years; *P* = .004). There was no significant difference in median (IQR) age of MyCode-identified male participants with vs without iron overload (65.2 [44.1-70.0] years vs 61.1 [42.2-71.4] years; *P* = .37) (eTable 3 in Supplement 2).

**Table 3.** Frequency of *HFE*-Associated Phenotypes in MyCode-Identified and Clinically Identified Participants

*HFE*-associated diagnosis, No. (%)	Females, No./total No. (%)		*P* value	OR (95% CI)	Males, No./total No. (%)		*P* value	OR (95% CI)
	MyCode identified	Clinically identified			MyCode identified	Clinically identified		
HH1 diagnosis	31/85 (36.5)	29/29 (100)	<.001^a^	NA	24/59 (40.7)	28/28 (100)	<.001^a^	NA
Iron overload, any	29/85 (34.1)	23/29 (79.3)	<.001^a^	0.14 (0.05-0.37)	24/59 (40.7)	19/28 (67.9)	.02^a^	0.32 (0.12-0.84)
Laboratory criteria: transferrin saturation ≥45% and serum ferritin ≥300 ng (male) or ≥200 ng (female)	29/85 (34.1)	21/29 (72.4)	<.001^a^	0.20 (0.08-0.50)	23/59 (39.0)	18/28 (64.3)	.03^a^	0.35 (0.14-0.90)
Imaging^b^	5/85 (5.9)	1/29 (3.4)	>.99^c^	1.75 (0.20-15.63)	6 /59 (10.2)	4/28 (14.3)	.72^c^	0.68 (0.18-2.63)
Other criteria^d^	2/85 (2.4)	9/29 (31.0)	<.001^c^^,^^a^	0.05 (0.01-0.27)	2/59 (3.4)	8/28 (28)	.001^a^	0.09 (0.02-0.45)
Heart disease^e^	10/85 (11.8)	5/29 (17.2)	.53^c^	0.64 (0.20-2.06)	8/59 (13.6)	6/28 (21.4)	.36^c^	0.58 (0.18-1.85)
Fibrosis in participants without HCV infection and AUD	2/75 (2.7)	6/26(23.1)	.003^c^^,^^a^	0.09 (0.02-0.49)	1/48 (2.1)	6/22 (27.3)	.003^c^^,^^a^	0.06 (0.01-0.51)
Cirrhosis in participants without HCV infection and AUD	0/75	3/26 (11.5)	.02^c^^,^^a^		0/48	1/22 (4.6)	.31^c^	NA
Chronic liver disease (all) in participants without HCV infection and AUD	30/75 (40.0)	15/26 (57.7)	.12	0.49 (0.20-1.21)	23/48 (47.9)	12/22 (54.6)	.61	0.77 (0.28-2.11)
NASH or NAFLD in participants without HCV infection and AUD	17/75 (22.7)	12/26 (46.2)	.02^a^	0.34 (0.13-0.88)	15/48 (31.2)	8/22 (36.4)	.67	0.80 (0.28-2.30)
Abnormal result from liver function study in participants without HCV infection and AUD^f^	9/75 (12.0)	9/26 (34.6)	.02^c^^,^^a^	0.26 (0.09)	9/48 (18.8)	7/22 (31.8)	.23	0.49 (0.16-1.57)
Other chronic liver disease in participants without HCV infection and AUD^g^	10/75 (13.3)	3/26 (11.5)	>.99^c^	1.18 (0.30-4.66)	4/48 (8.3)	3/22 (13.6)	.67^c^	0.58 (0.12-2.82)
Fibrosis in individuals with HCV infection and/or AUD	0/10	0/3	NA	NA	1/11 (9.1)	3/6 (50.0)	.10^c^	0.10 (0.01-1.35)
Cirrhosis in individuals with HCV infection and/or AUD	0/10	0/3	NA	NA	1/11 (9.1)	1/6 (16.7)	>.99^c^	0.50 (0.02-9.77)
Chronic liver disease (all) in participants without HCV infection and/or AUD	8/10 (80.0)	3/3 (100)	>.99^c^	NA	6/11 (54.6)	6/6 (100)	.10^c^	NA

Abbreviations: AUD, alcohol use disorder; EHR, electronic health record; HCV, hepatitis C virus; HH1, hereditary hemochromatosis type 1; MRI, magnetic resonance imaging; NA, not applicable; NAFLD, nonalcoholic fatty liver disease; NASH, nonalcoholic steatohepatitis; OR, odds ratio.

^a^ Statistically significant at α = .05.

^b^ Participants were considered to have evidence of iron overload on imaging when their radiology report indicated evidence of iron overload (eg, moderate hepatic iron deposition noted on liver MRI).

^c^ Calculated with Fisher exact test.

^d^ Participants with other evidence of iron overload included those with iron overload on liver biopsy or other clinical documentation of iron overload by a clinician in the EHR.

^e^ Evidence of cardiomyopathy and/or heart failure in the EHR.

^f^ Abnormal result from liver function study was defined as 2 or more aspartate aminotransferase or alanine aminotransferase levels flagged as elevated in the EHR at least 3 months apart without an intervening normal value.

^g^ Other chronic liver disease included any other documentation of a chronic liver disease such as a diagnosis of liver disease on the problem list.

After result disclosure, 5 of 144 MyCode-identified participants (3.5%) had a liver biopsy, all of which were attributed to the *HFE* result. Four of these 5 participants met criteria for laboratory iron overload prior to the procedure, and 1 had transferrin saturation and serum ferritin levels within normal ranges; the biopsy was prompted by a liver MRI demonstrating moderate to severe hepatic steatosis and hepatic iron concentration of 1.4 mg/g. Three of 144 participants underwent cardiac MRI after result disclosure, and 2 had the study in response to their genetic result and iron studies. Of these 3 participants, 2 had abnormal serum ferritin levels, 1 met laboratory criteria for iron overload, and 1 had the procedure performed for an unrelated indication (chemotherapy), although this participant had an abnormal serum ferritin result prior to imaging.

Thirty-six (69.2%) of the 52 MyCode-identified participants with laboratory^10,12^ or liver biopsy evidence of iron overload began phlebotomy (n = 35) or chelation (n = 1) after result disclosure. An additional 6 participants (11.5%) initiated phlebotomy after result disclosure without documented iron overload in the EHR. Two of these 6 participants received care outside of the Geisinger system; external records were not available to assess eligibility for intervention. Of the remaining 4 participants, 3 had abnormal serum ferritin result and 1 also had abnormal transferrin saturation level, although none met laboratory criteria for iron overload. All 4 participants had phlebotomy that was recommended by their treating hematologist.

### Comparisons Across 3 Participant Groups 

The highest serum ferritin, transferrin saturation, and serum iron values and lowest TIBC levels of MyCode-identified participants were compared with those of participants negative for p.Cys282Tyr homozygosity and were stratified by sex assigned at birth. Compared with participants negative for p.Cys282Tyr homozygosity, MyCode-identified participants had higher median (IQR) levels of serum ferritin (females: 165.0 [77.0-450.5] ng/mL vs 92.1 [42.0-197.3] ng/mL, *P* < .001; males: 714.6 [294.0-1037.0] ng/mL vs 222.7 [113.9-438.4] ng/mL, *P* < .001), transferrin saturation (females: 61.5 [36.0-80.0] μg/dL vs 26.0 [18.0-35.0] μg/dL, *P* < .001; males: 59.0 [49.0-95.0] μg/dL vs 29.0 [21.0-39.0] μg/dL, *P* < .001), and serum iron (females: 141.0 [97.0-166.0] μg/dL vs 92.1 [42.0-197.3] μg/dL, *P* < .001; males: 147.0 [124.0-192.0] μg/dL vs 87.0 [63.0-113.0] μg/dL, *P* < .001) and a lower median (IQR) TIBC level (females: 226.5 [197.0-257.5] μg/dL vs 286.0 [233.0-333.0] μg/dL, *P* < .001; males: 224.0 [189.0-259.0] μg/dL vs 251.0 [192.0-300.0] μg/dL, *P* = .02) (Figure 2; eTable 2 in Supplement 2). MyCode-identified participants had higher rates of laboratory iron overload (females: 34.1% vs 2.1%, *P* < .001; males: 39.0% vs 2.9%, *P* < .001) (eTable 4 in Supplement 2).

The frequency of *HFE*-associated phenotypes in MyCode-identified participants was compared with that in clinically identified participants and stratified by sex assigned at birth. MyCode-identified participants had a lower frequency of iron overload (females: 34.1% vs 79.3% [*P* < .001], OR, 0.14 [95% CI, 0.05-0.37]; males: 40.7% vs 67.9% [*P* = .02], OR, 0.32 [95% CI, 0.12-0.84]) and some liver-associated phenotypes (eg, fibrosis in individuals without HCV or AUD: females, 2.7% vs 23.1% [*P* = .003], OR, 0.09 [95% CI, 0.02-0.49]; males, 2.1% vs 27.3% [*P* = .003], OR, 0.06 [95% CI, 0.01-0.51]) (Table 3). A secondary analysis combining MyCode and clinically identified participants found that those with AUD or HCV infection were 3.74 times more likely to have chronic liver disease (23 of 30 [76.7%]) compared with those without (80 of 171 [46.8%]) (eTable 5 in Supplement 2). No participants had hepatocellular carcinoma. Of the 144 MyCode-identified participants, 18 (12.5%) had cardiomyopathy or heart failure. These phenotype rates did not differ from those of clinically identified participants. Age of onset of *HFE*-associated phenotypes in MyCode-identified participants compared with clinically identified participants is summarized in eTable 6 in Supplement 2.

## Discussion

This cross-sectional study provided insights into the implications of population genomic screening for *HFE* p.Cys282Tyr homozygosity. It confirmed that iron overload was underdiagnosed among those with positive p.Cys282Tyr homozygosity results, which has implications for preventing morbidity and mortality. Additionally, two-thirds of MyCode-identified participants had a relevant laboratory test after result disclosure, and most eligible participants proceeded with phlebotomy or chelation, demonstrating the ability of genomic screening to prompt relevant health behaviors.

Unlike previous studies,^13,14,15,16,30^ the present study included genetic result disclosure, which allowed for comparison of *HFE*-associated phenotypes in MyCode-identified participants vs clinically identified participants and those negative for p.Cys282Tyr homozygosity. These comparisons highlighted that genomic screening increased identification of iron overload while identifying participants with decreased disease severity compared with clinically identified participants. Given the limited postdisclosure period and the role of age in diagnosis of iron overload, future studies with a longer follow-up period are needed.

Even with conservative criteria for attributing behaviors to result disclosure, a sizable proportion of participants altered their care in response to the genetic results, suggesting the potential role of p.Cys282Tyr genomic screening. Over two-thirds of participants underwent a recommended laboratory test after disclosure, most of which were attributable to the genetic result. The proportion of participants engaging in risk management that was directly associated with result disclosure was likely higher. Laboratory tests lacking explicit documentation might have been prompted by result disclosure, as transferrin saturation and serum ferritin, while not uncommonly ordered, are not routine screening tests.

Clinical evaluation after identification of iron overload is an intermediate outcome associated with the recommended intervention of therapeutic phlebotomy or chelation. A total of 69.2% of eligible participants initiated an iron-lowering intervention. Six participants initiated phlebotomy without EHR evidence of iron overload, and thus, may not have met guidelines for this intervention. However, their treating clinician may have had additional information that was not captured in the EHR to prompt treatment.

Although additional studies are needed to explore the implications of p.Cys282Tyr screening for the prevention of end organ damage, preventing ESLD is important. A meta-analysis identified substantial impairment in quality of life in individuals with ESLD.^31^ Patients with ESLD and their families also experience substantial financial burden and distress associated with worse health outcomes.^32^ These findings support the importance of early interventions, including consideration of genomic screening, that prevent organ damage.

In addition to illustrating the ability of genomic screening to identify individuals with iron overload and to guide care, these results begin to inform the potential implementation of genomic screening. MyCode-identified participants had evidence of iron overload at the time of screening. MyCode-identified participants had a median age of 56.6 years, and the data suggest that screening at a younger age may identify individuals with iron overload earlier and enable appropriate intervention to prevent subsequent organ damage. Additionally, individuals with evidence of AUD or HCV infection were more likely to have chronic liver disease compared with individuals without these risk factors. Although further research is needed to elucidate the additive effects of the genetic and environmental risks for liver injury, these results suggest genomic screening may have added benefit for those with other risk factors for chronic liver disease.

Consistent with a previous study, we also speculate that genomic screening for HH1 susceptibility would be cost-effective,^33^ particularly if added to a program that is already screening for other disorders.^34^ First, genetic testing costs are decreasing, and identifying iron overload through laboratory testing and the associated intervention if iron overload is identified (phlebotomy) is low cost. Second, phlebotomy is known to correct iron overload and resulting organ damage if initiated before irreversibility. Based on our study results, uptake of testing and interventions is substantial. Third, the costs of organ damage, such as cirrhosis and cardiomyopathy, are high. We propose testing these speculations through rigorous cost-effectiveness studies, including decision modeling using recommended best practices.^35^

### Limitations

This study has several limitations. First, phenotype and management behaviors were extracted from the Geisinger EHR. Thus, capture of diagnoses and health behaviors was limited if a patient received care outside of the Geisinger system. This study was completed in a single health system; additional studies are needed to ensure the generalizability of results across systems and the population. Second, the study relied on process and intermediate outcomes to determine clinical utility. Doing so was unavoidable given that health outcomes of interest, such as liver fibrosis and cardiomyopathy, take years to develop. Thus, it is necessary to rely on a chain of evidence demonstrating that improved intermediate outcomes play a role in improved health outcomes. In another study, there was robust evidence that intervention in individuals with clinically apparent iron overload was effective in preventing end organ damage.^36^ This finding, coupled with the modest transient harms associated with therapeutic phlebotomy,^30^ supports the provision of intervention to individuals with presymptomatic iron overload.^13^

## Conclusions

This cross-sectional study identifies the potential benefit of population genomic screening for *HFE* p.Cys282Tyr homozygosity. While more research is needed on the implications of genomic screening for health outcomes and cost-effectiveness, findings of this study support the return of p.Cys282Tyr homozygosity results to patients as an actionable secondary finding and its potential inclusion in population screening.

## Notes

Supplement 1.
eTable 1.

Supplement 2. **eMethods.** HFE Abstraction Manual **eTable 1.** See Supplement 1 **eTable 2.** Laboratory Values in Females and Males Homozygous for p.Cys282Tyr Identified Via MyCode and Compared to Participants Negative for p.Cys282Tyr Homozygosity **eTable 3.** Effect of Iron Overload on Age at Results Disclosure in MyCode-Identified Participants **eTable 4.** Comparison of Laboratory Iron Overload in MyCode Identified Participants Compared to Participants Negative for p.Cys282Tyr Homozygosity **eTable 5.** Comparison of Liver Disease Frequency in MyCode and Clinically Identified p.Cys282Tyr Homozygotes With and Without Liver Disease Risk Factors **eTable 6.** Age of Onset of HFE-Associated Phenotypes in HFE C282Y Homozygotes Identified Via MyCode and in Clinically Ascertained Participants

Supplement 3.
Data Sharing Statement

## References

[1] Online Mendelian Inheritance in Man (OMIM). Johns Hopkins University; 2016. September 8, 2016. Accessed June 7, 2021. https://www.omim.org/entry/235200

[2] GenBank. National Library of Medicine, National Center for Biotechnology Information. Accession No. NM_000410.4, Homo sapiens homeostatic iron regulator (HFE), transcript variant 1, mRNA. Accessed June 18, 2021. https://www.ncbi.nlm.nih.gov/nuccore/NM_000410

[3] Online Mendelian Inheritance in Man (OMIM). Johns Hopkins University; 2022. May 19, 2022. Accessed February 20, 2022. https://www.omim.org/entry/613609

[4] Olynyk JK, Ramm GA. Hemochromatosis. N Engl J Med. 2022;387(23):2159-2170. doi:10.1056/NEJMra2119758 36477033

[5] ClinVar. National Library of Medicine, National Center for Biotechnology Information. VCV000000009.24. Accessed June 7, 2021. https://www.ncbi.nlm.nih.gov/clinvar/variation/9/

[6] gnomAD browser. Broad Institute. Single nucleotide variant:6-26093141-G-A(GRCh37). Accessed June 7, 2021. https://gnomad.broadinstitute.org/variant/6-26093141-G-A

[7] Powell LW, Seckington RC, Deugnier Y. Haemochromatosis. Lancet. 2016;388(10045):706-716. doi:10.1016/S0140-6736(15)01315-X 26975792

[8] Whitlock EP, Garlitz BA, Harris EL, Beil TL, Smith PR. Screening for hereditary hemochromatosis: a systematic review for the U.S. Preventive Services Task Force. Ann Intern Med. 2006;145(3):209-223. doi:10.7326/0003-4819-145-3-200608010-00009 16880463

[9] Qaseem A, Aronson M, Fitterman N, Snow V, Weiss KB, Owens DK; Clinical Efficacy Assessment Subcommittee of the American College of Physicians. Screening for hereditary hemochromatosis: a clinical practice guideline from the American College of Physicians. Ann Intern Med. 2005;143(7):517-521. doi:10.7326/0003-4819-143-7-200510040-00010 16204164

[10] Bacon BR, Adams PC, Kowdley KV, Powell LW, Tavill AS; American Association for the Study of Liver Diseases. Diagnosis and management of hemochromatosis: 2011 practice guideline by the American Association for the Study of Liver Diseases. Hepatology. 2011;54(1):328-343. doi:10.1002/hep.24330 21452290 PMC3149125

[11] Dotson WD, Kolor K, Khoury MJ, Grosse SD. Tier 1 guidelines on family-based screening for hereditary hemochromatosis. March 12, 2021. Accessed December 1, 2022. https://blogs.cdc.gov/genomics/2021/03/12/tier-1-guidelines/

[12] Kowdley KV, Brown KE, Ahn J, Sundaram V. ACG Clinical Guideline: hereditary hemochromatosis. Am J Gastroenterol. 2019;114(8):1202-1218. doi:10.14309/ajg.0000000000000315 31335359

[13] Grosse SD, Gurrin LC, Bertalli NA, Allen KJ. Clinical penetrance in hereditary hemochromatosis: estimates of the cumulative incidence of severe liver disease among HFE C282Y homozygotes. Genet Med. 2018;20(4):383-389. doi:10.1038/gim.2017.121 28771247 PMC5797490

[14] Pilling LC, Tamosauskaite J, Jones G, . Common conditions associated with hereditary haemochromatosis genetic variants: cohort study in UK Biobank. BMJ. 2019;364:k5222. doi:10.1136/bmj.k5222 30651232 PMC6334179

[15] Atkins JL, Pilling LC, Masoli JAH, . Association of hemochromatosis HFE p.C282Y homozygosity with hepatic malignancy. JAMA. 2020;324(20):2048-2057. doi:10.1001/jama.2020.21566 33231665 PMC7686863

[16] Tamosauskaite J, Atkins JL, Pilling LC, . Hereditary hemochromatosis associations with frailty, sarcopenia and chronic pain: evidence from 200,975 older UK Biobank participants. J Gerontol A Biol Sci Med Sci. 2019;74(3):337-342. doi:10.1093/gerona/gly270 30657865 PMC6376086

[17] Miller DT, Lee K, Chung WK, ; ACMG Secondary Findings Working Group. ACMG SF v3.0 list for reporting of secondary findings in clinical exome and genome sequencing: a policy statement of the American College of Medical Genetics and Genomics (ACMG). Genet Med. 2021;23(8):1381-1390. doi:10.1038/s41436-021-01172-3 34012068 PMC13097145

[18] Carey DJ, Fetterolf SN, Davis FD, . The Geisinger MyCode community health initiative: an electronic health record-linked biobank for precision medicine research. Genet Med. 2016;18(9):906-913. doi:10.1038/gim.2015.187 26866580 PMC4981567

[19] MyCode Scorecard. 2 Million Geisinger patients. July 1, 2023. Accessed July 20, 2023. https://www.geisinger.org/-/media/OneGeisinger/pdfs/ghs/research/mycode/mycode-scorecard.pdf?la=en

[20] Buchanan AH, Lester Kirchner H, Schwartz MLB, . Clinical outcomes of a genomic screening program for actionable genetic conditions. Genet Med. 2020;22(11):1874-1882. doi:10.1038/s41436-020-0876-4 32601386 PMC7605431

[21] Dewey FE, Murray MF, Overton JD, . Distribution and clinical impact of functional variants in 50,726 whole-exome sequences from the DiscovEHR study. Science. 2016;354(6319):aaf6814. doi:10.1126/science.aaf6814 28008009

[22] Williams MS, Buchanan AH, Davis FD, . Patient-centered precision health in a learning health care system: Geisinger’s genomic medicine experience. Health Aff (Millwood). 2018;37(5):757-764. doi:10.1377/hlthaff.2017.1557 29733722

[23] Schwartz MLB, McCormick CZ, Lazzeri AL, . A model for Genome-First care: returning secondary genomic findings to participants and their healthcare providers in a large research cohort. Am J Hum Genet. 2018;103(3):328-337. doi:10.1016/j.ajhg.2018.07.009 30100086 PMC6128218

[24] Faucett WA, Davis FD. How Geisinger made the case for an institutional duty to return genomic results to biobank participants. Appl Transl Genom. 2016;8:33-35. doi:10.1016/j.atg.2016.01.003 27047758 PMC4796707

[25] Kalia SS, Adelman K, Bale SJ, . Recommendations for reporting of secondary findings in clinical exome and genome sequencing, 2016 update (ACMG SF v2.0): a policy statement of the American College of Medical Genetics and Genomics. Genet Med. 2017;19(2):249-255. doi:10.1038/gim.2016.190 27854360

[26] Kelly MA, Leader JB, Wain KE, . Leveraging population-based exome screening to impact clinical care: The evolution of variant assessment in the Geisinger MyCode research project. Am J Med Genet C Semin Med Genet. 2021;187(1):83-94. doi:10.1002/ajmg.c.31887 33576083

[27] Gallego CJ, Burt A, Sundaresan AS, . Penetrance of hemochromatosis in HFE genotypes resulting in p.Cys282Tyr and p.[Cys282Tyr];[His63Asp] in the eMERGE Network. Am J Hum Genet. 2015;97(4):512-520. doi:10.1016/j.ajhg.2015.08.008 26365338 PMC4596892

[28] Harris PA, Taylor R, Minor BL, ; REDCap Consortium. The REDCap consortium: building an international community of software platform partners. J Biomed Inform. 2019;95:103208. doi:10.1016/j.jbi.2019.103208 31078660 PMC7254481

[29] Harris PA, Taylor R, Thielke R, Payne J, Gonzalez N, Conde JG. Research electronic data capture (REDCap)–a metadata-driven methodology and workflow process for providing translational research informatics support. J Biomed Inform. 2009;42(2):377-381. doi:10.1016/j.jbi.2008.08.010 18929686 PMC2700030

[30] Ong SY, Gurrin LC, Dolling L, . Reduction of body iron in HFE-related haemochromatosis and moderate iron overload (Mi-Iron): a multicentre, participant-blinded, randomised controlled trial. Lancet Haematol. 2017;4(12):e607-e614. doi:10.1016/S2352-3026(17)30214-4 29195602

[31] Peng JK, Hepgul N, Higginson IJ, Gao W. Symptom prevalence and quality of life of patients with end-stage liver disease: a systematic review and meta-analysis. Palliat Med. 2019;33(1):24-36. doi:10.1177/0269216318807051 30345878 PMC6291907

[32] Ufere NN, Satapathy N, Philpotts L, Lai JC, Serper M. Financial burden in adults with chronic liver disease: a scoping review. Liver Transpl. 2022;28(12):1920-1935. doi:10.1002/lt.26514 35644920 PMC9669101

[33] de Graaff B, Neil A, Si L, . Cost-effectiveness of different population screening strategies for hereditary haemochromatosis in Australia. Appl Health Econ Health Policy. 2017;15(4):521-534. doi:10.1007/s40258-016-0297-3 28035629

[34] Guzauskas GF, Garbett S, Zhou Z, . Population genomic screening for three common hereditary conditions: a cost-effectiveness analysis. Ann Intern Med. 2023;176(5):585-595. doi:10.7326/M22-0846 37155986 PMC11791829

[35] United States Department of Veterans Affairs. Health Services Research and Development Service QUERI Economic Analysis Guidelines. December 2009. Accessed August 6, 2023. https://www.herc.research.va.gov/files/MPDF_303.pdf.

[36] Vanclooster A, Wollersheim H, Vanhaecht K, Swinkels D, Aertgeerts B, Cassiman D; Haemochromatosis working group. Key-interventions derived from three evidence based guidelines for management and follow-up of patients with HFE haemochromatosis. BMC Health Serv Res. 2016;16(1):573. doi:10.1186/s12913-016-1835-2 27733158 PMC5062877

