# Supplementary material for: Testing and Management of Iron Overload After Genetic Screening–Identified Hemochromatosis
Source: JAMA Netw Open. 2023 Oct 23;6(10):e2338995. doi: 10.1001/jamanetworkopen.2023.38995 (PMC10594145; doi:10.1001/jamanetworkopen.2023.38995)
Supplement: Supplement 2. — eMethods. HFE Abstraction Manual eTable 1. See Supplement 1 eTable 2. Laboratory Values in Females and Males Homozygous for p.Cys282Tyr Identified Via MyCode and Compared to Participants Negative for p.Cys282Tyr Homozygosity eTable 3. Effect of Iron Overload on Age at Results Disclosure in MyCode-Identified Participants eTable 4. Comparison of Laboratory Iron Overload in MyCode Identified Participants Compared to Participants Negative for p.Cys282Tyr Homozygosity eTable 5. Comparison of Liver Disease Frequency in MyCode and Clinically Identified p.Cys282Tyr Homozygotes With and Without Liver Disease Risk Factors eTable 6. Age of Onset of HFE-Associated Phenotypes in HFE C282Y Homozygotes Identified Via MyCode and in Clinically Ascertained Participants [file jamanetwopen-e2338995-s002.pdf]

Supplemental Online Content

Savatt JM, Johns A, Schwartz MLB, et al. Testing and management of iron overload after genetic screening–identified hemochromatosis. *JAMA Netw Open*. 2023;6(10):e2338995. doi:10.1001/jamanetworkopen.2023.38995

eMethods. HFE Abstraction Manual

eTable 1. See Supplement 1

eTable 2. Laboratory Values in Females and Males Homozygous for p.Cys282Tyr Identified Via MyCode and Compared to Participants Negative for p.Cys282Tyr Homozygosity

eTable 3. Effect of Iron Overload on Age at Results Disclosure in MyCode-Identified Participants

eTable 4. Comparison of Laboratory Iron Overload in MyCode Identified Participants Compared to Participants Negative for p.Cys282Tyr Homozygosity

eTable 5. Comparison of Liver Disease Frequency in MyCode and Clinically Identified p.Cys282Tyr Homozygotes With and Without Liver Disease Risk Factors

eTable 6. Age of Onset of HFE-Associated Phenotypes in HFE C282Y Homozygotes Identified Via MyCode and in Clinically Ascertained Participants

This supplemental material has been provided by the authors to give readers additional information about their work.

© 2023 Savatt JM et al. *JAMA Network Open*

eMethods. HFE Abstraction Manual

1 GENERAL NOTES 3

2 DEMOGRAPHICS 7

3 SOCIAL HISTORY 10

4 CLINICAL: FIRST DIAGNOSIS 12

5 CLINICAL: LAB VALUES 16

6 CLINICAL: SECONDARY CAUSES 18

7 CLINICAL: HFE-RELATED DIAGNOSIS 19

8 CLINICAL: SERVICES 22

9 FAMILY HISTORY 24

© 2023 Savatt JM et al. *JAMA Network Open*

1 General notes

- Each question in the abstraction manual is noted with a numbered heading (e.g. “2.1 Patient deceased?”. Immediately under the heading is the question type (e.g. “yes/no”).
- Manual chart review instructions are marked with ***Epic***.
- All patient history in Epic is used for the chart review (there is no specified lookback limit).
- RoR = Return of Results
- ICD = International Classification of Disease Code
- CPT = Current Procedural Terminology

2 Manual chart review

- If you need to reference a specific question in the REDCap survey, use the "Question ID" listed at the bottom of each question (e.g. "2019-08-20.105").

- DO NOT use data from other health systems that comes from Care Everywhere. The IRB protocol for the GSC Registry does not include external data sources.

- **Problem list.** When looking for diagnoses in the problem list, check "Show past problems"

- **Medical history.** The list of diagnoses under History > Medical overlaps with the problem list but is **not** identical. You should look in both this and the problem list to avoid missing something.

- When correcting an automatically abstracted data, add in the "Notes" field where the correct information was found in the chart.

- **ICD codes are not searchable in Epic.** Chart abstractors will typically use keyword searches instead.
    - The exception to this is if a clinician manually puts an ICD code into a note. But this is not guaranteed – there may be many problem list entries that are never added to notes.

- Epic search does not show anything from the problem list unless it was specifically pulled into a note by a clinician.

- **Epic search may miss results for some keywords.** This appears to be because Epic searches automatically for synonyms (e.g. if you search for "tumor", results for "neoplasm" may also appear), but sometimes this process breaks. **Do not rely solely on searching for a term** until you manually verify that Epic's search behaves as you expect it to.

- Epic search results at Geisinger typically do not include pathology reports, but do include imaging.

© 2023 Savatt JM et al. *JAMA Network Open*

2.1 Standard response options

- **Date.** This question is for verifying a date from the automatic extraction. Context from the automatic extract may also be included for convenience (e.g. ICD code for a diagnosis date).

  Response options are:
  1. Date: _______
  2. Year only: _______
  3. Age: _______
  4. In chart without date
  5. Not in chart
  6. Missing (a part of the chart where this information is stored is missing)
  7. Not applicable (question doesn't make sense for patient)

- **Date relative to RoR.** Same as "date" but with additional options related to RoR:
  1. Date: _______
  2. Year only: _______
  3. Age: _______
  4. Chart indicates before RoR, but without exact date
  5. Chart indicates only after RoR, but without exact date
  6. In chart without date
  7. Not in chart
  8. Missing (a part of the chart where this information is stored is missing)
  9. Not applicable (question doesn't make sense for patient)

Notes on dates:

For parts of the chart that include a date that is not a diagnosis date (e.g. "added date" for the problem list), use "in chart without date" or one of the "no exact date" options.

If just year and month is available, assume the 1st day of the month.

For free text fields:
- Missing = 8888
- Not applicable = 9999

2.2 Automatic data extraction

Data are automatically extracted from PIDB, a deidentified clinical data warehouse. PIDB extracts are updated every quarter.

2.2.1 ICD code wildcards

ICD codes ending with "%" indicate a "wildcard".

This means that in addition to an exact match, a more specific code with the same prefix qualifies.

© 2023 Savatt JM et al. *JAMA Network Open*

Examples:

- “F10%” would match “F10”, “F10.1”, “F10.10”, … “F10.2”, …
- “F10.1%” would match “F10.1”, “F10.10”, “F10.11”, …
    - But this would **not** match “F10.2”

© 2023 Savatt JM et al. *JAMA Network Open*

3 Demographics

3.1 Patient deceased?

Yes or no

Epic

- Look for flag when opening chart.
- Header bar will be gray

3.2 Date of birth

Date field

Epic Look in header

3.3 First encounter

Date field

Purpose: estimate how long Geisinger's electronic medical records go back for the patient

Epic Chart Review > Encounters. Sort by "when" ascending and click "load all records" button. Record the earliest available record (no other criteria).

© 2023 Savatt JM et al. *JAMA Network Open*

3.4 Last encounter

Date field

Purpose: see how behind the automated extract is from Epic.

> If the automated extract is behind the last encounter in Epic, check for additional data in this gap for each applicable survey question **in the entire chart review**.

Epic

- Chart Review > Encounters. Sort by "when", recent to oldest.
- Choose the most recent encounter **with evidence of new clinical data or interaction with the patient.**
    - For example, office visits, telephone encounters (where the patient answers), and MyGeisinger encounters where a patient read and responded all count.
    - Ignore any encounters that are not accompanied by new clinical data or interaction with the patient (e.g. MyCode orders, medication refills).
    - The purpose of this is to identify the gap between PIDB and Epic, so we don't want to artificially increase this gap.

3.5 Sex

Free text *(note: we expect this and the other demographics fields to be automatically extracted with high accuracy; free text is just for corrections)*

Epic

1. Check header to see if there is difference between sex in DOB field and "Gender identity".
2. If there is, click "Gender Identity" to reveal and record sex at birth in REDCap.
    a. Automatic extract is based on legal sex.

Automated extract is most likely based on legal sex. We want sex at birth, however.

3.6 Race

Free text

Epic Demographics > Clinical information

Epic has 3 race fields, which are combined into 1 question in REDCap. We expect the automated extract to be highly accurate for this field, but if you need to fill it in, you don't need to indicate values for the 2nd and 3rd fields if blank.

3.7 Ethnicity

Free text

Epic Demographics > Clinical information

© 2023 Savatt JM et al. *JAMA Network Open*

3.8 Height and Weight

Numeric, plus dropdowns for units

Epic Review flowsheets > BMI

Note that header does not display height and weight unless it was captured at the latest encounter.

If manually entered, we will assume height and weight are accurate as of the last encounter date in Epic.

© 2023 Savatt JM et al. *JAMA Network Open*

4 Social history

All automatically extracted data uses the most recently available data. See the “Last PIDB encounter” date at the top of the page for the most recent possible date for the automatic extract.

4.1 Smoking status

Free text

Epic History > Substance & Sexual activity > “Smoking Status” field

Automated extract:
Value of SOC_HX_SMOKE_STTS at most recent encounter

4.2 Smoking tobacco quit date

Date

Epic History > Substance & Sexual activity > “Quit Date” field

Automated extract:
Most recent value of SOC_HX_SMOKE_STTS indicating no smoking tobacco use, with history of smoking in SOC_HX_SMOKE_STTS.

4.3 Active alcohol drinker

Yes/no

Epic History > Substance & Sexual activity > “Alcohol use” field

Automated extraction:
Most recent social history update has SOC_HX_ALC_YN (flag to indicate use of alcohol at last encounter)

4.4 Intravenous (IV) drug use

Yes/no

Epic

Any of the following:

- History > Substance & Sexual activity > Substance Use.
    - IV checked
    - Drug commonly used IV is checked ("heroin", "fentanyl")
- Problem list
    - Mention of IV drug use or one of the drugs commonly used IV (mentioned above)

Collected as IV drug use may be related to increased risk for hepatitis C.

Automatic extraction:

None (not available in PIDB).

4.5 Alcohol abuse/dependence, earliest date noted

Date

Epic

- Search for:
    - Alcohol abuse
    - Alcohol dependence
- Problem list/medical history: "alcohol abuse" or "alcohol dependence"

Automatic extraction:

ICD codes	Description
F10%	Alcohol related disorders
303.9%	Other and unspecified alcohol dependence

© 2023 Savatt JM et al. *JAMA Network Open*

5 Clinical: first diagnosis

The purpose of this section is to determine whether HFE-related diagnoses were made before RoR. To accomplish this, we collect the **date of the first diagnosis** for each condition.

Response options for each question are: standard “verify date relative to RoR”’

Criteria is “clear diagnosis”. A note indicating “likely” or “favoring” does not count.

Additionally, we may collect what the diagnosis was based off of (in the form of checkboxes for various types of medical information).

5.1 HFE C282Y/C282Y genotyping

Date relative to RoR

Epic

1. Search “HFE” and “hemochromatosis”, and look for lab reports
2. Results Review > Genetics > HFE C282Y (or similar)
3. Results Review > Laboratory Results > Anemia, and look for “HFE” or “hemochromatosis”

Automatic extraction:
Not currently abstracted automatically, but we might be able to add this in in the future.

5.2 Hemochromatosis diagnosis

Date relative to RoR

Epic

- Problem list > check “Show past problems”
- “Medical History” (History > Medical)
- Chart review > Encounters > Filters > Diagnosis/Impressions; look for HFE
- Search for ***any*** of the following:
  - Any mention of “hemochromatosis”
  - Do not count “biallelic mutation of HFE gene” – the Genetic Counselors are adding this in for each patient

Automatic extraction:
Any of the following diagnosis:

ICD codes	Description
E83.119	Hemochromatosis, unspecified
275.01	Hereditary hemochromatosis
275.02	Hemochromatosis due to repeated red blood cell transfusions
275.03	Other hemochromatosis

5.3 Iron Overload

Date relative to RoR

Epic

Any of the following:

- Search for “iron” and see if anything related to disorders of iron metabolism comes up.
- Search for “Fibroscan” (term for ultrasound of liver) and look at results for “iron overload”
- Look on the problem list and see if disorders of iron metabolism on there
- Pathology or imaging report that clearly indicates “iron overload”.

If yes: What medical information was the diagnosis of iron overload based off of?

- Clinical
- Imaging (Ultrasound, MRI, CT)
- Unknown

Automatic extraction:

Any of the following diagnosis:

ICD codes	Description
275.0	Disorders of iron metabolism
275.09	Other disorders of iron metabolism
E83.10	Disorder of iron metabolism, unspecified

Or, meeting these lab criteria:

- Ever transferrin saturation greater than 45 percent**, and**
- Ever serum ferritin (search for “FERRITIN” in Results Review)
    - Men: greater than 300 ng/mL
    - Women: greater than 200 ng/mL
- Note that the above **do not** need to occur at the same time.

© 2023 Savatt JM et al. *JAMA Network Open*

5.4 Liver cirrhosis diagnosis

Date relative to RoR

Epic

Any of the following:

- Problem list/problem history: includes “cirrhosis”
- Search for “cirrhosis”, “cirrhotic liver”, or “fibrotic liver”
    - Diagnosis may be from imaging of abdomen (ultrasound, CT, MRI)
- Pathology
    1. Look at past surgical history for “biopsy liver” to find relevant dates. Often this will be a “wedge liver biopsy”, but other kinds of biopsies can be used (needle, core, ultrasound guided, etc.).
    2. For each date in #1, go to Results Review > Pathology, and look at that pathology result for “cirrhosis” or “stage 4 fibrosis”. **Note on next question if diagnosis is from pathology report.**

If yes: Liver Cirrhosis type/source

- Alcoholic cirrhosis
- Non-alcoholic Steatohepatitis (NAFL/NASH)
- Other cirrhosis type (including chronic viral)
- Unknown cirrhosis type
- Source: from pathology report
- Source: from imaging report

Automatic extraction:

Any of the following diagnosis:

ICD code	Description
K70.2	Alcoholic fibrosis and sclerosis of liver
K70.3%	Alcoholic cirrhosis of liver
K71.7	Toxic liver disease with fibrosis and cirrhosis of liver
K74%	Fibrosis and cirrhosis of liver
K76.1	Chronic passive congestion of liver
571.2	Alcoholic cirrhosis of liver
571.5	Cirrhosis of liver without mention of alcohol
571.6	Biliary cirrhosis

© 2023 Savatt JM et al. *JAMA Network Open*

5.5 Other chronic liver disease

Date relative to RoR

Epic

Any of the following:

- Lab results (look under "Liver" to see both AST and ALT the same time)
  - Persistent elevation of AST **or** ALT for >3 months
    - "Elevation" means: flagged as high by the lab
    - We need either:
      - 2+ flags for AST in a row spaced >3 months apart
      - 2+ flags for ALT in a row spaced >3 months apart
      - Flagged values **cannot** be separated by a normal value to count

Only continue to search criteria if the patient does not meet lab criteria.

- Search for "liver" or "hepat" or "transaminitis"
  - We are specifically interested in the presence of any chronic liver inflammation, so look for:
    - "hepatitis",
    - "transaminitis",
    - inflammatory liver disease,
    - chronic liver disease,
    - or "fatty liver".

Automatic extraction:

Lab criteria from above, or any of the following diagnosis:

ICD codes	Exception	Description
K70.0		alcoholic fatty liver
K70.1%		alcoholic hepatitis
K71%	Except K71.1%	toxic liver disease (except with hepatic necrosis, which is used in previous question)
K73%		chronic hepatitis, not elsewhere classified
K75%	Except K75.0 and K75.1	other inflammatory liver diseases (except for abscess or phlebitis of portal vein)
K76.0		fatty liver, not elsewhere classified
K77%		liver disorders in diseases classified elsewhere
K76.9		liver disease, unspecified
571.0		alcoholic fatty liver
**571.3**		alcoholic liver damage, unspecified
571.4		chronic hepatitis
571.8		other chronic nonalcoholic liver disease
571.9		unspecified chronic liver disease without mention of alcohol
573%	Except 573.4, 573.5	other liver disorders

© 2023 Savatt JM et al. *JAMA Network Open*

6 Clinical: lab values

In this section, we are collecting the following for each of the lab values below:

1. Extreme value ever (with collected date)
    a. This is the highest value, except for TIBC which is the lowest value.
    b. Was this value flagged by the lab? Possible options are “high”, “low”, or “abnormal”.
2. Earliest collected date before RoR (no lab value)
3. First collected date on or after RoR (no lab value)
4. **Only if not successfully collected after RoR:** date ordered (but not completed), after RoR only
    a. ***Epic*** Do a global search for the lab name, or go to Chart Review > Labs. Filter by "Order" and then search within orders for the lab name.

Collected date is used for available values because that is much easier to access than the ordered or resulted date.

6.1 Transferrin saturation

[See top of section for question type]

Epic Laboratory results > Anemia > Iron Screen > Transferrin Sat %, + flag status if flag is high

Automatic extraction:

- Lab value for transferrin saturation (TRANSFERRIN SAT) [%]

6.2 Ferritin level

[See top of section for question type]

Epic Search for “FERRITIN” in Results Review, + flag status if flag is high

Automatic extraction:

- Lab value for ferritin (FERRITIN) [ng/mL]

6.3 Iron serum level

[See top of section for question type]

Epic Laboratory results > Anemia > Iron, or Laboratory results > Anemia > Iron Screen; Lab test called “IRON” , + flag status if flag is high

Automatic extraction:

- Lab value for serum iron (IRON) [mcg/dL]

6.4 TIBC level

[See top of section for question type]

Epic Laboratory results > Anemia > Iron Screen > “Iron binding cap” , + flag status if flag is low

Automatic extraction:

- Lab value for total iron binding capacity (IRON BINDING CAP) [mcg/dL]

© 2023 Savatt JM et al. *JAMA Network Open*

- Relevant extreme value is lowest, not highest

6.5 Liver panel

[See top of section for question type; note values are not collected, just the presence/absence of values.]

Epic Results review > Liver, or Results review > General chemistry

Look for an aspartate aminotransferase (AST) value present, and an alanine aminotransferase (ALT) value present. They do not need to be ordered at the same time.

For prior to RoR, both AST and ALT **must** be present before RoR. If they aren't ordered at the same time, use the date of the later one.

Automatic extraction:

- Lab value presence for aspartate aminotransferase (AST) [u/l] at any time, and
- Lab value presence for alanine aminotransferase (ALT) [u/l] at any time

© 2023 Savatt JM et al. *JAMA Network Open*

7 Clinical: secondary causes

The purpose of this section is to determine if secondary causes occurred before RoR.

Criteria is "clear diagnosis". A note indicating "likely" or "favoring" does not count.

7.1 Any secondary causes of hemochromatosis prior to RoR

Date relative to RoR; separate dates are collected for each condition listed below.

Epic Search for the conditions listed in the "Automatic extraction" section, plus look in problem list and medical history sections.

Also search for the following terms, which may help find the conditions below (but are not directly attributable to any specific condition):

- Hemoglobinopathy
- Hemolytic anemia

For "recurrent transfusion", search for "transfusion" and apply the criteria below:

- Multiple transfusions in a 12-month period that are *not* for a onetime cause (e.g. trauma, surgery), or
- ≥10 separate transfusions for any reason in the history.

(These are not official clinical criteria; just the criteria for this chart review.)

Automatic extraction:

Condition	ICD Code(s)	ICD description
Thalassemia	D56.9<br>282.4%	Thalassemia, unspecified
Pyruvate Kinase deficiency	D55.2 (non-specific)<br>282.3	Anemia due to disorders of glycolytic enzymes<br>Other hemolytic anemias due to enzyme deficiency
X-Linked Sideroblastic Anemia	D64.0<br>285.0	Hereditary sideroblastic anemia
Recurrent transfusion	E83.111<br>275.02	Hemochromatosis due to red blood cell transfusions
Sickle Cell Anemia	D57%<br>282.6%	Sickle-cell disorders
Hereditary Spherocytosis	D58.0<br>282.0<br>282.1<br>282.8<br>282.9	Hereditary Spherocytosis
Congenital dyserythropoietic anemia	D64.4<br>285.8	Congenital dyserythropoietic anemia

© 2023 Savatt JM et al. *JAMA Network Open*

8 Clinical: HFE-related diagnosis

The purpose of this section is to determine if HFE-related disease findings were established before or after RoR.

We are asking for the **earliest diagnosis date** for each of the conditions below.

If multiple factors in a given category appear in the chart, choose the earliest one.

Criteria is “clear diagnosis”. A note indicating “likely” or “favoring” does not count.

8.1 Arthritis diagnosis

Date relative to RoR

Epic Search for the conditions listed below, plus look in problem list and medical history sections.

Automatic extraction:

Any of the following diagnosis:

ICD code	Description
M19.04%	Primary osteoarthritis, hand
M19.24%	Secondary osteoarthritis, hand
M15%	Polyosteoarthritis
715%	Osteoarthrosis and allied disorders

8.2 Hepatocellular carcinoma diagnosis

Date relative to RoR

Epic

- Search for “cancer”, “carcinoma”, “malignant”, “neoplasm” and look for results related to the liver
- Also look in problem list and medical history sections for the same terms, related to liver.

Automatic extraction:

Any of the following diagnosis:

ICD code	Description
C22%	Malignant neoplasm of liver and intrahepatic bile ducts
155.0	Malignant neoplasm of liver, primary

© 2023 Savatt JM et al. *JAMA Network Open*

8.3 Heart disease (e.g. heart arrhythmias, heart failure) diagnosis

Date relative to RoR

Epic

- Search for the conditions listed below, plus look in problem list and medical history sections.
- Also search for “Atrioventricular Block” or “AV Block”, which are related to cardiac arrhythmias.

Automatic extraction:
Any of the following diagnosis:

ICD code	Description
I42%<br>425%	Cardiomyopathy
I49.5	Other cardiac arrhythmias
I49.9	Cardiac arrhythmia, unspecified
I50%<br>428%	Heart failure
427.8<br>427.9	Cardiac dysrhythmia

8.4 MRI or cardiology note indicating heart disease is a consequence of HFE

Yes/no

Only if 7.3 (heart disease) is “yes”:

Epic Search for “HFE” and “hemochromatosis”; look for:

- Mention of iron overload/ excess in MRI, or
- Explicit statement by cardiology about HFE related heart disease

Automatic extraction:
Not available.

8.5 Type 2 diabetes mellitus diagnosis

Date relative to RoR

Epic Search for “diabetes” and look for results for Type 2 diabetes, plus look in problem list and medical history sections.

Automatic extraction:
Any of the following diagnosis:

ICD code	Description
E11%	Type 2 diabetes mellitus
ICD9 codes mentioning Type 2 diabetes: 250.00, 250.02, 250.10, 250.12, 250.20, 250.22, 250.30, 250.32, 250.40, 250.42, 250.50, 250.52, 250.60, 250.62, 250.70, 250.72, 250.80, 250.82, 250.90, 250.92	

© 2023 Savatt JM et al. *JAMA Network Open*

8.6 Fatigue diagnosis
Date relative to RoR

Epic Look in problem list and medical history sections.

Automatic extraction:
Any of the following diagnosis:

ICD code	Description
R53.8%	Other malaise and fatigue
780.7%	Other malaise and fatigue

8.7 Impotence diagnosis
Date relative to RoR

Epic Search for “erectile” and “impotence”, plus look in problem list and medical history sections.

Automatic extraction:
Any of the following diagnosis:

ICD code	Description
N52%	Male erectile dysfunction
607.84	Male erectile dysfunction, unspecified

© 2023 Savatt JM et al. *JAMA Network Open*

9 Clinical: services

The purpose of this section is to determine whether these services were utilized before RoR or not.

Note that if a service was utilized before RoR, we will **not** capture whether it was used after RoR.

9.1 Liver biopsy

Date relative to RoR

Epic

- Search for “biopsy” and look for anything related to the liver.
- Look at past surgical history for “biopsy liver”. Often this will be a “wedge liver biopsy”, but other kinds of biopsies can be used (needle, core, ultrasound guided, etc.).

Automatic extraction:

Any CPT code listed below.

CPT code	Description
47379	Laparoscopic Procedures on the Liver
47001	Liver biopsy
47100	Biopsy of liver, wedge

9.2 Phlebotomy

Date relative to RoR

Epic Filter labs by “phleb” and look for “therapeutic phlebotomy”

Automatic extraction:

Any CPT code listed below.

CPT code	Description
99195	Therapeutic phlebotomy

9.3 Chelation therapy

Date relative to RoR

Note: This is rarely used for treating HFE.

Epic Search for the following drug names:

- Deferasirox
- Deferoxamine
- Deferiprone

Presence of any indicates chelation therapy.

Automatic extraction:
Any HCPCS code listed below.

HCPCS code	Description
J0895	INJ,DEFEROXAMINE MESYLATE,500
S9355	Home infusion therapy, chelation therapy

9.4 Cardiac MRI
Date relative to RoR

Epic Either:
1. Chart Review > Cardiac Studies > MRI, or
2. Results Review > Cardiology > MRI

Automatic extraction:
Any CPT code listed below.

CPT code	Description
75557	Cardiac mri for morph
75559	Cardiac mri w/stress img
75561	Cardiac mri for morph w/dye
75563	Card mri w/stress img & dye
75565	Card mri veloc flow mapping
75552 through 75556	Old CPT codes for Cardiac MRI

10 Family history

Data collected:
For all the diseases of interest (hemochromatosis, colorectal cancer, breast cancer, liver cancer), the following is collected for each:

1. Yes/no: any family history?
2. Checkbox: family members affected
3. Dropdown: total number of affected relatives (response options are 1, 2, 3, 4, 5+)
4. Dropdown: youngest age of diagnosis for any family member (response options are 5 year age buckets)

Epic

- Look in History > Family
    - For relatives with the icon that indicates that there is additional free text, click the icon to view details.
- Search for “cancer” and “hemochromatosis”, look for mentions of family history
- Search for "family history" and "fhx", look for mentions of the diseases below
- We are specifically interested in colorectal, breast and liver cancer
- Search for GC note. They take a screenshot often and put that in Epic.
- Any mention of genotype or phenotype counts.

Automatic extraction:
Will not be abstracted automatically; not reliable from PIDB.

© 2023 Savatt JM et al. *JAMA Network Open*

eTable 2. Laboratory Values in Females and Males Homozygous for p.Cys282Tyr Identified Via MyCode and Compared to[a] Participants Negative for p.Cys282Tyr Homozygosity

Laboratory Study	Median Extreme Value (IQR, Range) Females Identified Via MyCode	Median Extreme Value (IQR, Range) Females Negative for p.Cys282Tyr Homozygosity	P-Value, Odds Ratio, Confidence Interval	Median Extreme Value (IQR, Range) Males Identified Via MyCode	Median Extreme Value (IQR, Range) Males Negative for p.Cys282Tyr Homozygosity	P-Value, Odds Ratio, Confidence Interval
**Serum Iron Highest Value (ug/dL)** Female's Normal Range 33-151 ug/dL Male's Normal Range 45-176 ug/dL	141.0 (97.0-166.0, 30.0-239.0.0) n=61	81.0 (58.0-108.0, 0.5-826.0) n=23388	**<0.0001***	147.0 (124.0-192.0, 22.0-307.0) n=37	87.0 (63.0- 113.0, 5.0- 668.0) n=13880	**<0.0001***
**Ferritin Highest Value (ng/mL)** Female's Normal Range 13-150 ng/mL Male's Normal Range 30-400 ng/mL	165.0 (77.0-450.5, 4.4-2682.0) n=67	92.1 (42.0-197.3, 1.0- 100000.0) n=22279	**<0.0001***	714.6 (294.0-1037.0, 25.3-2856.0) n=37	222.7 (113.9-438.4, 1.0-100000.0) n=11736	**<0.0001***
**Transferrin Highest Value (%)** Normal Range 15-55%	61.5 (36.0-80.0, 8.0-100.0) n=60	26.0 (18.0- 35.0, 1.0- 109.0) n=18495	**<0.0001***	59.0 (49.0-95.0, 24.0-100.0) n=35	29.0 (21.0- 39.0, 2.0- 145.0) n=11400	**<0.0001***
**Total Iron Binding Capacity Lowest Value (ug/dL)** Normal Range 250-425 ug/dL	226.5 (197.0-257.5, 74.0-325.0) n=60	286.0 (233.0-333.0, 1.0 - 787.0) n=21380	**<0.0001***	224.0 (189.0-259.0, 52.0-323.0) n=35	251.0 (192.0-300.0, 1.0- 614.0) n=13002	**0.0187***

[a] Compared using Wilcoxon rank sum test *denotes statistical significance at an alpha of 0.05.

© 2023 Savatt JM et al. *JAMA Network Open*

eTable 3. Effect of Iron Overload on Age at Results Disclosure in MyCode-Identified Participants

Iron Overload phenotypes	Median Age of Females Identified Via MyCode with Iron Overload. Median, IQR	Median Age of Female Identified Via MyCode without Iron Overload. Median, IQR	Median Female Identified Via MyCode Age Comparison, P value	Median Age of Males Identified Via MyCode with Iron Overload. Median, IQR	Median Age of Males Identified Via MyCode without Iron Overload. Median, IQR	Median Male Identified Via MyCode Age Comparisons, P-value
**Iron Overload ANY**	61.3 (51.1-69.9)	46.2 (35.1-67.9)	**0.0036***	65.2 (44.1-70.0)	61.1 (42.2-71.4)	0.3727
**Iron Overload Lab Criteria[a]**	60.9 (50.9-68.8)	47.0 (35.3-68.5)	**0.0108***	65.8 (44.2-70.1)	59.9 (43.1-70.9)	0.3274
**Iron Overload Imaging[b]**	59.1 (47.9-80.9)	54.0 (40.2-68.4)	0.4389	68.7 (67.5-77.2)	62.3 (43.1-68.7)	**0.0272***
**Iron Overload Other Criteria[c]**	67.2 (58.3-73.8)	52.7 (39.0-67.9)	0.0503	67.8 (59.6-69.1)	63.0 (43.5-70.9)	0.2284

[a]Lab Criteria (Transferrin ≥ 45% AND Serum Ferritin ≥ 300ng (Male) or ≥ 200ng (Female)) [b]Participants were considered to have evidence of iron overload on imaging when their radiology report indicated evidence of iron overload (e.g., moderate hepatic iron deposition noted on liver MRI). [c]Participants with other evidence of iron overload included those with iron overload on liver biopsy or other clinical documentation of iron overload by a provider in the EHR. *denotes statistical significance at an alpha of 0.05.

© 2023 Savatt JM et al. *JAMA Network Open*

eTable 4. Comparison of Laboratory Iron Overload in MyCode Identified Participants Compared to[a] Participants Negative for p.Cys282Tyr Homozygosity

HFE-related diagnosis	Females Detected through MyCode GSC n=85	Females Negative for p.Cys282Tyr Homozygosity n=52994	P-Value, Odds Ratio (Confidence Interval)	Males Detected through MyCode GSC n=59	Males with Negative for p.Cys282Tyr Homozygosity n=33301	P-Value, Odds Ratio, Confidence Interval
**Laboratory Iron Overload** (Transferrin ≥ 45% AND Serum Ferritin ≥ 300ng (Male) or ≥ 200ng (Female))	29 (34.1%)	1120 (2.1%)	**<0.0001*, 23.98 (15.26, 37.70)**	23 (39.0%)	962 (2.9%)	**<0.0001*, 21.48 (12.68, 36.38)**

[a]Compared using Fisher's exact test * denotes statistical significance

© 2023 Savatt JM et al. *JAMA Network Open*

eTable 5. Comparison of Liver Disease Frequency in MyCode and Clinically Identified p.Cys282Tyr Homozygotes with and without Liver Disease Risk Factors[a]

**HFE-related diagnosis, No., (%)**	***HFE*-related liver diagnosis in individuals without HepC[c] and/or Alcohol Abuse (N=171)**	***HFE*-related liver diagnosis in individuals with HepC[c] and/or Alcohol Abuse (N=30)**	**Total (N=201)**	**P-Value, Odds Ratio, Confidence Interval**
**Liver Disease**	81 (47.4%)	25 (83.3%)	106 (52.7%)	**0.0003*,** 5.56 (2.03, 15.19)
Fibrosis	15 (8.8%)	4 (13.3%)	19 (9.4%)	0.4952[d], 1.60 (0.49, 5.20)
Cirrhosis	4 (2.3%)	2 (6.7%)	6 (3.0%)	0.2202[d], 2.98 (0.52, 17.06)
Chronic Liver Disease (all)[b]	80 (46.8%)	23 (76.7%)	103 (51.2%)	**0.0025*,** 3.74 (1.52, 9.17)

[a]Liver disease risk factors include alcohol abuse and hepatitis C infection. [b]Chronic liver disease includes nonalcoholic steatohepatitis and nonalcoholic fatty liver disease (NASH/NAFLD), abnormal result from liver function study was defined as two or more Aspartate Aminotransferase (AST) or Alanine Aminotransferase (ALT) flagged as elevated in the EHR at least three months apart without an intervening normal value, and any other documentation of a chronic liver disease such as a diagnosis of liver disease on problem list [c]HepC -- hepatitis C viral infection. [d]Fisher's Exact test. *denotes statistical significance at an alpha of 0.05.

© 2023 Savatt JM et al. *JAMA Network Open*

eTable 6. Age of Onset of HFE-Associated Phenotypes in *HFE* C282Y Homozygotes Identified Via MyCode and in Clinically Ascertained Participants

**HFE-related diagnosis, Age in years (IQR)**	**Median Age in Years (IQR) in Females Detected through MyCode GSC n=85**	**Median Age in Years (IQR) in Females with Previous Diagnosis n=29**	**P-Value**	**Median Age in Years (IQR) in Males Detected through MyCode GSC n=59**	**Median Age in Years (IQR) in Males with Previous Diagnosis n=28**	**P-Value**	**Median Age in Years (IQR) in all Detected through MyCode GSC n=144**	**Median Age in Years (IQR) in Previous Diagnosis n=57**	**P-Value**
**Hemochromatosis Diagnosis**	60.1 (49.7-71.9) n=31	51.4 (35.5-60.7) n=29	**0.0065***	58.8 (42.6-71.7) n=24	55.4 (43.6-60.6) n=28	0.3261	60.1 (44.5-71.9) n=55	54.3 (38.8-60.7) n=57	**0.0065***
**Iron Overload (any)**	61.5 (52.7-68.4) n=29	58.7 (47.6-66.0) n=23	0.1507	63.6 (44.1-71.7) n=24	61.3 (55.2-65.1) n=19	0.5655	61.6 (48.3-70.1) n=53	60.6 (49.9-65.5) n=42	0.1936
Lab Criteria (Transferrin ≥ 45% AND Serum Ferritin ≥ 300ng (Male) or ≥ 200ng (Female))	61.5 (53.3-68.5) n=29	58.7 (47.6-65.5) n=21	0.1879	64.4 (43.9-73.3) n=23	61.8 (55.5-65.1) n=28	0.5902	62.1 (49.2-70.6) n=52	61.2 (49.9-65.5) n=49	0.2498
Imaging[a]	58.8 (48.0-80.5) n=5	59.3 (59.3-59.3) n=1	>0.9999	71.1 (55.1-77.8)	64.6 (63.7-71.4)	1.0000	68.5 (48.0-78.1)	64.6 (62.8-64.6)	0.9098
Other Criteria[b]	60.6 (52.7-68.4) n=2	53.9 (51.5-66.4) n=9	0.5557	69.3 (68.3-70.2) n=2	60.7 (47.8-64.7) n=8	0.1510	68.4 (60.5-69.3) n=4	60.0 (51.5-66.0) n=17	0.0975
**Heart Disease[c]**	70.7 (55.1-78.3) n=10	65.3 (63.9-73.5) n=5	0.5815	67.5 (57.5-69.0) n=8	63.2 (57.3-80.9) n=6	0.8465	68.3 (55.1-76.0) n=18	64.6 (57.3-79.6) n=11	0.6694

© 2023 Savatt JM et al. *JAMA Network Open*

HFE-related diagnosis, Age in years (IQR)	Median Age in Years (IQR) in Females Detected through MyCode GSC n=85	Median Age in Years (IQR) in Females with Previous Diagnosis n=29	P-Value	Median Age in Years (IQR) in Males Detected through MyCode GSC n=59	Median Age in Years (IQR) in Males with Previous Diagnosis n=28	P-Value	Median Age in Years (IQR) in all Detected through MyCode GSC n=144	Median Age in Years (IQR) in Previous Diagnosis n=57	P-Value
**Liver Disease in those without HepC[d] and/or Alcohol Abuse**	52.1 (42.0-65.1) n=30	59.5 (46.8-66.0) n=15	0.6387	48.9 (43.5-68.1) n=23	53.9 (48.5-65.3) n=13	0.8434	51.6 (42.8-65.4) n=53	55.5 (47.7-65.8) n=28	0.6160
Fibrosis	35.8 (21.1-50.5) n=2	58.7 (51.5-66.8) n=6	0.1336	49.2 (49.2-49.2) n=1	62.4 (57.1-68.5) n=6	0.4533	49.2 (21.1-50.5) n=3	59.8 (54.3-67.7) n=12	0.0513
Cirrhosis	-	69.5 (67.5-70.6) n=3	-	-	75.7 (75.7-75.7) n=1	-	-	70.1 (68.5-73.2) n=4	-
Chronic Liver Disease[e]	52.1 (42.0-65.1) n=30	59.5 (46.8-66.0) n=15	0.6387	48.9 (43.5-68.1) n=23	53.1 (43.0-64.7) n=12	1.0000	51.6 (42.8-65.4) n=53	53.9 (46.8-65.7) n=27	0.7307
**Liver Disease in those with HepC[d] and/or Alcohol Abuse**	39.7 (29.9-49.3) n=8	51.6 (35.8-51.7) n=3	0.4750	43.5 (30.0-62.0) n=8	55.3 (55.1-59.3) n=6	0.7469	39.7 (29.9-54.6) n=16	55.1 (51.6-55.3) n=9	0.2949
Fibrosis	-	-	-	68.3 (68.3-68.3) n=1	59.3 (55.2-64.5) n=3	0.3711	68.3 (68.3-68.3) n=1	59.3 (55.2-64.5) n=3	0.3711
Cirrhosis	-	-	-	53.5 (53.5-53.5) n=1	55.1 (55.1-55.1) n=1	_	53.5 (53.5-53.5) n=1	55.1 (55.1-55.1) n=1	-
Chronic Liver Disease[e]	39.7 (29.9-49.3) n=8	51.6 (35.8-51.7) n=3	0.4750	32.9 (27.8-55.7) n=6	55.3 (55.1-61.3) n=6	0.5752	34.0 (28.8-52.7) n=14	55.1 (51.6-55.3) n=9	0.1564

[a]Participants were considered to have evidence of iron overload on imaging when their radiology report indicated evidence of iron overload (e.g., moderate hepatic iron deposition noted on liver MRI). [b]Participants with other evidence of iron overload included those with iron overload on liver biopsy or other clinical documentation of iron overload by a provider in the EHR [c]Cardiomyopathy and/or heart failure [d]HepC hepatitis C viral infection [e]Chronic liver disease includes nonalcoholic steatohepatitis and nonalcoholic fatty liver disease (NASH/NAFLD), abnormal result from liver function study was defined as two or more Aspartate Aminotransferase (AST) or Alanine Aminotransferase (ALT) flagged as elevated in the EHR at least three months apart without an intervening normal value, and any other documentation of a chronic liver disease such as a diagnosis of liver disease on problem list *denotes statistical significance at an alpha of 0.05. Blank cells did not have any participants with that phenotype.

© 2023 Savatt JM et al. *JAMA Network Open*
