# Supplementary material for: Testing and Management of Iron Overload After Genetic Screening–Identified Hemochromatosis
Source: JAMA Netw Open. 2023 Oct 23;6(10):e2338995. doi: 10.1001/jamanetworkopen.2023.38995 (PMC10594145; doi:10.1001/jamanetworkopen.2023.38995)
Supplement: Supplement 3. — Data Sharing Statement [file jamanetwopen-e2338995-s003.pdf]

Data Sharing Statement

Data
Data available: No

Additional Information
Explanation for why data not available: Some or all datasets generated during and/or analyzed during the current study are not publicly available but are available from the corresponding author upon reasonable request.
